# Ductal Carcinoma *In Situ* Biology, Biomarkers, and Diagnosis

**DOI:** 10.3389/fonc.2017.00248

**Published:** 2017-10-23

**Authors:** Kylie L. Gorringe, Stephen B. Fox

**Affiliations:** ^1^Cancer Genomics Program, Peter MacCallum Cancer Centre, Melbourne, VIC, Australia; ^2^The Sir Peter MacCallum Department of Oncology, University of Melbourne, Parkville, VIC, Australia; ^3^Department of Pathology, Peter MacCallum Cancer Centre, Melbourne, VIC, Australia

**Keywords:** ductal carcinoma *in situ*, invasive breast cancer, progression, recurrence, biomarkers, microenvironment, gene

## Abstract

Ductal carcinoma *in situ* (DCIS) is an often-diagnosed breast disease and a known, non-obligate, precursor to invasive breast carcinoma. In this review, we explore the clinical and pathological features of DCIS, fundamental elements of DCIS biology including gene expression and genetic events, the relationship of DCIS with recurrence and invasive breast cancer, and the interaction of DCIS with the microenvironment. We also survey how these various elements are being used to solve the clinical conundrum of how to optimally treat a disease that has potential to progress, and yet is also likely over-treated in a significant proportion of cases.

## Introduction

**Figure 1:**
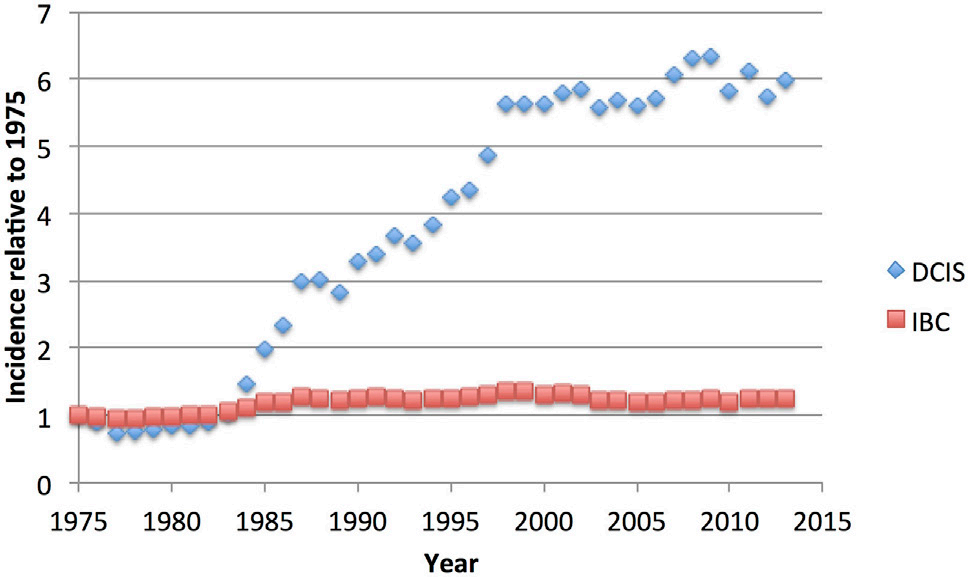
US National Cancer Institute Surveillance, Epidemiology, and End Results (SEER) age-adjusted incidence of ductal carcinoma *in situ* (DCIS) compared to invasive breast cancer (IBC), relative to the rate of each observed in 1975, showing the dramatic increase in DCIS cases, without noticeable decrease in IBC cases (6).

Implementation of widespread mammographic screening has led to an increase in diagnosis of breast tumors such as ductal carcinoma *in situ* (DCIS, Figure 1). Previously uncommon, DCIS now comprises ~20% of all breast carcinoma diagnoses (1). DCIS shares many of the epidemiological risk factors as invasive breast cancer (IBC) including age, family history, parity, and some other hormonal factors and high mammographic density (2). Weaker risk factors such as alcohol consumption and high body mass index have been inconsistently associated with DCIS risk. The genetic risk factors are also similar: *BRCA1* and *BRCA2* mutation carriers develop DCIS more frequently and at an earlier age than the general population (3, 4) and are significantly more likely to have occult DCIS in prophylactic mastectomies than age-matched non-carriers from autopsy studies (5).

Because of its nature as a potential precursor for invasive breast carcinoma, excision of DCIS is recommended, but the lack of a concomitant decrease in the IBC diagnosis rate (Figure 1) suggests that much DCIS is being over-treated and would never progress to invasive disease nor give rise to any morbidity. Indeed, autopsy studies indicate that occult DCIS exists in ~9% of women (range 0–15%) (7). In the few studies with small numbers of DCIS where misdiagnosis led to omission of surgery, 14–53% of women developed IBC over 30 years (8–10). A recent meta-analysis placed the 15-year invasive recurrence rate after surgery alone for DCIS at 28% and breast cancer–specific mortality at 18% (11). Thus, while most DCIS must be treated to prevent invasive disease, there is a substantial proportion that may never become invasive. The difficulty clinicians grapple with is how to discriminate between high- and low-risk entities, and how to best advise their patients. Given unclear guidelines, some patients elect for more aggressive treatment than is necessary, such as mastectomy with axillary node dissection or even bilateral mastectomy (12, 13). Here, we review the current state of understanding of DCIS biology, pathology, treatment, and its relationship to invasive disease.

## Diagnosis and Pathology of DCIS

Ductal carcinoma *in situ* is a proliferation of atypical epithelial cells that is contained within the lumen of the breast ductal system. Nowadays, it is usually detected in the context of a mammographic screening program, but can occasionally (more commonly in pre-screening times) present as a palpable lump or with other physical symptoms like nipple discharge (14). Approximately 8% of core needle biopsies are initially diagnosed as DCIS (15), and this diagnosis is confirmed in ~74% of cases after excision. A recent meta-analysis found that under-diagnosis on core biopsy (a diagnosis of DCIS on biopsy changed to invasive disease after excision) was associated with large tumor size, palpable mass, a mammographic mass lesion, use of image guidance other than stereotactic, and high mammographic density (15).

The degree of cellular atypia is determined histologically whereby three grade levels are assigned (low, intermediate, and high) on the basis of the degree of nuclear atypia (16). High-grade tumors show marked nuclear pleomorphism, large nucleic size, conspicuous mitoses, and irregular chromatin. In contrast, low nuclear grade refers to monotonous nuclei of small size more akin to normal luminal epithelial cell size and only occasional nucleoli and mitoses. Intermediate grade is defined as neither low nor high grade, which may account for its poor inter-observer reproducibility (17). The highest grade present is reported, although grade heterogeneity has been observed in 12–50% of cases (18, 19). High-grade tumors, which represent 42–53% of DCIS cases (20–23), are considered a high risk factor for recurrence (22, 24–26) and breast cancer-specific mortality (27), although some studies do not show such an effect (21, 28). The presence of high grade in a biopsy correlates with a higher probability of the presence of invasive disease (15).

**Figure 2:**
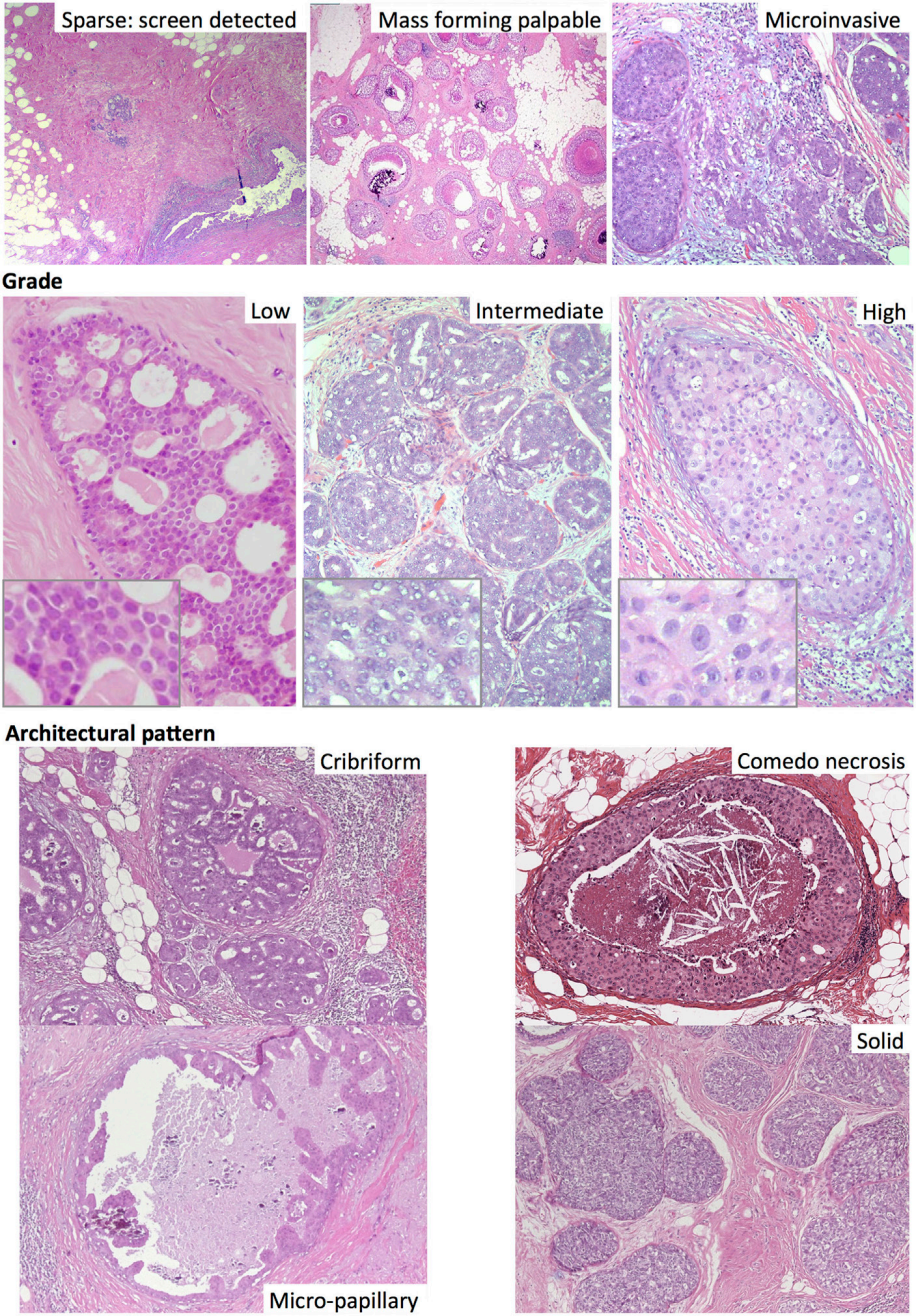
Different subtypes of ductal carcinoma *in situ*, including by mode of detection (top). Haematoxylin and eosin images.

In addition to nuclear atypia, a range of different architectural patterns are observed, including cribriform, solid, comedo (central necrosis), micropapillary, and papillary (Figure 2). Multiple patterns are often observed within the same tumor (46–62% of cases) (19, 29), which may explain the low level of concordance of studies using these categories as prognostic markers. The prognostic value of these architectural features has been found to be limited; comedo necrosis is associated with high grade and worse breast cancer-specific survival (27) but only inconsistently with recurrence (26, 28). The increase in incidence of DCIS after the introduction of mammographic screening has been more strongly associated with an increase in the non-comedo subtypes (30).

## Overview of Screening and Effect on DCIS Diagnosis Rate and Mortality

Although the rate of DCIS diagnosis has risen in the mammographic era, mortality rates from DCIS have fallen. A Swedish study found that the standard mortality ratio after DCIS fell from 5.29 in cases diagnosed 1980–1990 to 3.30 for cases 2000–2011 (31). Screen-detected DCIS have been shown to have a lower rate of invasive recurrence, and lower overall mortality (24, 32). This improved mortality is likely due in part to earlier detection, with more recently diagnosed DCIS being smaller (30) but also due to the shift in type, with a reduction in the proportion that have poor prognostic features such as high grade or comedo necrosis. These features support the concept of over-diagnosis, and yet a comparison of screening units in the UK found that when screening units with different sensitivity of detection are compared, those with a higher DCIS detection rate had a lower interval IBC rate (33). This result suggests that screening can in fact prevent invasive disease.

## Treatment of DCIS

Ductal carcinoma *in situ* treatment currently is variable, and depends on the preferences of surgeon and patient (see below). Almost all women will elect to have surgery, and this is usually a wide-local excision (WLE), though a percentage will have a mastectomy if the DCIS is high grade and extensive or if the patient prefers. Radiotherapy (RT) is offered after WLE and clinical trial data show a 4–5-fold reduction in ipsilateral recurrences (19–31%) compared with contralateral tumors (4–7%) at 10 years when treated with surgery alone (34–36). After treatment with RT, the gap narrows to ~2-fold (7–20% ipsi- vs. 3–8% contralateral). Endocrine therapy in estrogen receptor (ER) positive tumors reduces the contralateral and ipsilateral recurrences to a similar degree (34). The effect of endocrine therapy on ipsilateral recurrence is minimal if RT is also applied, suggesting that RT alone can be effective in killing residual disease cells. Long-term outcome data for DCIS show that regardless of treatment, breast cancer-specific mortality is very low (1.5–2% at 10 years up to 6.3% at 30 years) (23, 31).

The variation in treatment selected among physicians, by country and by treatment center can be dramatic (23). For example, a recent series from Australia (1994–2005) reported that 85% of women with DCIS had WLE only, 9% had mastectomy, just 6% were given RT after WLE, and 26% were also treated with tamoxifen (37). At the other extreme, 81% of patients received a mastectomy in a cohort in China, with less than 20% receiving RT but a surprising 43% receiving chemotherapy (38). Many patients also received hormonal therapy in this cohort (62%). In contrast, a large study from the Netherlands (1989–2004) treated 48% of women with mastectomy, 26% with WLE only, and 26% with WLE + RT (20). None were given tamoxifen. In the US, analysis of Surveillance, Epidemiology, and End Results (SEER) data indicated that the most recent treatment choices (2010) were WLE + RT (47%), mastectomy (28%), and WLE only (22%) (23), similar to a large Australian/New Zealand cohort from 2004 to 2009 (39). Treatment trends have changed over time, with generally fewer mastectomies [although a rise in bilateral mastectomies in young women has been recently noted in the US (23)], and increasing rates of RT. Although national guidelines can influence the choice of therapy, this variation is in great part due to the uncertainty around what constitutes a “high-risk” DCIS, i.e., a DCIS at high risk of recurrence and/or progression to invasive carcinoma requiring RT or mastectomy.

One tool that has been developed to assist with treatment decision is the Van Nuys Prognostic Index (VNPI) (40). Features associated with high risk of recurrence such as tumor size, margin status, grade, and patient age are combined in an index ranging from 4 to 12 that directs the practitioner to a suggested therapy (Table 1). Patients with a low score show no significant benefit from RT, in contrast to those with an intermediate or high score. The VNPI has been tested in a number of retrospective studies, but has yet to be used in a clinical trial context. Gilleard et al. observed the score to be significantly associated with recurrence-free survival after WLE only, with the low risk group having no recurrences (41). They also found that including age did not improve the prediction. However, MacAusland et al. did not find the index to be of prognostic value within 5 years after WLE ± tamoxifen (42). Other studies found the index to have prognostic power, but to lack utility in advising treatment, as most patients (59–79%) were placed into the “intermediate” grouping (43, 44).

**Table 1:** The Van Nuys Prognostic Index and recommendations for treatment.

Feature	Score 1	Score 2	Score 3
Size (mm)	≤15	16–40	>40
Margins (mm)	≥10	1–9	<1
Grade and necrosis	Low or intermediate without necrosis	Low or intermediate with necrosis	High grade with/without necrosis
Age (years)	>60	40–60	<40

	**Low score (4–6)**	**Intermediate (7–9)**	**High (10–12)**

% patients	32.6%	56.7%	10.8%
Treatment recommendation	Wide-local excision (WLE)	WLE + radiotherapy (RT)	Mastectomy
10 year recurrence-free survival^a^	97%	73%	34%
10 year breast cancer-specific survival	100%	98%	98%

*^a^After WLE ± RT, mastectomy excluded (40)*.

A similar score (45) incorporating grade, size, and age was tested on USA SEER data (for which margin status was not collected) and found a significant association with recurrence and also breast cancer-specific mortality (46). The latter study also showed that cases with a low score did not benefit from RT, and those with an intermediate score had only a limited benefit from RT.

More recently, a 10-feature nomogram was developed to assist with risk prediction after DCIS diagnosis (47), which incorporated age, family history, presentation, treatment, grade, margins, and, interestingly, the number of excisions. This latter feature was included despite not being predictive in a univariate analysis, yet three or more excisions led to an increased risk of recurrence in a multivariate model (HR 1.68). The number of excisions is rarely investigated in studies of DCIS and recurrence, possibly because of the difficulty in collecting such data. A later update from the same group at Memorial Sloan-Kettering Cancer Center showed that the number of excisions, along with margin width, was only predictive in a WLE-only group (48). This result is consistent with the idea that ipsilateral recurrences arise due to residual disease in the breast after surgery, which RT (and also endocrine therapy for ER+ disease) can alleviate.

Despite the many years these scoring tools have existed, and are apparently used by clinicians as a basis for discussion with patients, there does not appear to be any prospective validation of their utility. Attempts at validation using retrospective cohorts have had mixed results (49–52), and may be strongly influenced by the disparate cohorts available, in terms of treatment selection and completeness/accuracy of the data inputs. In a recent review (53), a DCIS decision tree was proposed to stratify patients for treatment, in which as yet hypothetical molecular markers were included. This strategy was employed to principally help discriminate the intermediate risk group in the VPNI, for whom treatment could vary from WLE only (for those with score 7, clear margins and good prognostic molecular markers) to mastectomy (for those with score 8/9, close or involved margins and poor prognostic molecular features). However, powerful biomarkers of recurrence for DCIS have yet to be determined.

## Molecular Features of DCIS

Invasive breast cancer can be categorized into a number of different subtypes based on molecular features, including immunohistochemical (IHC) markers, genetic features, and gene expression profiles. The most fundamental of these categories is related to the hormonal status of the tumor. Historically, DCIS has not been routinely evaluated for ER status, but research studies have found that the proportion of ER positivity at 62–76% (22, 23, 25, 54) is similar to that observed in IBC (55, 56). ER status is not currently used prognostically for DCIS, but current guidelines in the US indicate endocrine therapy for ER positive cases after WLE (57), and rates of ER testing have increased in recent years (58). Cancer registry data in the US suggests that at least 39% of women receive endocrine therapy (1). However, in the UK, NICE guidelines do not recommend endocrine therapy for DCIS (59), thus it is rarely prescribed.

### Genetic Events in DCIS

Genomic analysis of DCIS has been limited by the availability of fresh-frozen tissue resources, since the small average size means there is little left over after tissue requirements for clinical pathology have been met. In recent years, however, advancements in technology have meant that genome-wide approaches using formalin-fixed paraffin-embedded tissues from diagnostic material have been increasingly applied to DCIS. There are two main avenues of research: first, analyzing DCIS when observed in the same breast as IBC (“synchronous” or “mixed” DCIS) whereby the two components are compared for differences that may relate to invasive progression, and second, examining DCIS in the absence of invasive disease (“pure” DCIS).

The analysis of synchronous DCIS has found that despite their restriction to the ductal system, the genetic and expression profiles of these cells are remarkably similar to invasive disease. Early loss of heterozygosity (LOH) studies found high levels of allelic concordance in mixed DCIS/IBC components (60–62). An exome analysis of five mixed DCIS found that copy number and mutations had very high concordance levels between DCIS and IBC components in all cases (63). Similarly, genome-wide copy number analysis found that 18/21 mixed DCIS were clonally related to the invasive component, although some genetic heterogeneity was observed, which included regions recurrently present in the invasive but not matched DCIS (64). These differences included gains at known drivers such as *CCND1* and *MYC*. In some cases, an existing copy number gain in the DCIS was present at higher amplitude in the IBC region. This result was supported by a FISH analysis of amplified regions in synchronous DCIS/IBC showing increased amplicon level in the IBC (65).

A recent single-cell sequencing study of two mixed DCIS found some intriguing relationships (66). One HER2 positive case showed substantial intra-tumor heterogeneity, yet the DCIS and invasive cells were represented in all the different subclones, possibly suggesting an early acquisition of invasive potential and subsequent parallel evolution. Such a progression pathway could be occurring in DCIS with multiple foci of microinvasion, which can be observed particularly in large DCIS (67). In contrast, single-cell sequencing of an ER positive case showed evidence of a genetic bottleneck, whereby only one of the four DCIS subclones was closely related to the invasive cells (which showed low heterogeneity). Thus, there may be more than one possible mechanism of evolving an invasive phenotype.

Pure DCIS shows many of the same genetic events as mixed DCIS and IBC; however, overall the number of copy number changes is lower in pure DCIS (65, 68). There have as yet been too few genome-wide mutation studies of DCIS for a definitive comparison to IBC on mutation load and diversity. One targeted gene sequencing study found that all 20 DCIS studied had at least one mutated driver gene comparable with IBC (69); however, an exome analysis of high-grade DCIS found that a number of cases did not contain any drivers (70). Another small exome analysis comparing six pure DCIS with five mixed DCIS found that while individual pure DCIS each had a driver mutation, overall they had fewer mutations and copy number changes than mixed DCIS (63), which tended to have multiple drivers.

One of the key molecular differences between DCIS and IBC is the prevalence of *ERBB2* amplification. In IBC, HER2 positivity rates in population-based samples are ~14% (71). HER2 testing is not routinely done on DCIS cases, as anti-HER2 therapies are not employed, but the proportion reported in DCIS is consistently higher than IBC, ranging 18–56% with higher rates in high-grade DCIS, depending on the cohort (22, 25, 56, 72, 73). In addition, HER2 positivity may be a prognostic factor in DCIS predicting recurrence as DCIS but not as invasive cancer (22, 25, 68). Thus, *ERBB2* amplification alone may be insufficient for invasive progression and may even indicate a DCIS less likely to progress to invasion. Additional genetic events may be required for progression and whatever these changes are, they not only lead to invasion, but also to a very aggressive IBC subtype, an intriguing paradox.

One contributing event to invasive progression could be *TP53* mutation, as studies have consistently shown that *TP53* mutations are less frequent in pure DCIS (15% on average, 0–32%) (63, 69, 70, 74–80) than IBC (27–37%) (81, 82). In contrast, *PIK3CA* mutations appear to be similarly frequent in DCIS (24% on average, 17–55%) (63, 64, 69, 70, 74, 83–85) as to IBC (25–36%) (81, 82, 86, 87), although interestingly, several reports have noted the presence of *PIK3CA* mutation in the DCIS component of mixed DCIS/IBC but absent in the IBC component (64, 83). Some of these cases also had copy number data suggestive of either non-clonality or very early clonal divergence (64, 83). One study has suggested that *GATA3* mutations could be more common in DCIS (69), though this remains to be validated in other cohorts.

Correlations of mutation or copy number with features of DCIS have found that, similar to IBC, ER positivity is associated with *PIK3CA* mutation (69, 84), and also *GATA3* mutation (69). *TP53* mutation is associated with high grade and HER2 positivity (69, 76, 88), as well as a higher level of genomic copy number alteration (69). Genome-wide copy number changes and LOH events are more common in high-grade DCIS (68, 89–93), with specific increases seen for loss of 17p and gains of *ERBB2* and *MYC* (68, 92, 94–96). However, as in IBC, low-grade DCIS has frequent gain of 1q and loss of 16q (68, 92, 94). ER negative tumors have more copy number changes than ER positive, both overall and at specific loci (e.g., 8q gain, 5q loss, 15q loss), although ER positive tumors have more 16q losses (68). An integrated gene expression and copy number analysis found that DCIS have similar “integrated cluster” membership to IBC (93), and similarly, the breast cancer “intrinsic subtypes” correlated with genetic features such as *TP53* mutation frequency and copy number profiles (68, 69, 88).

### Expression Analysis of DCIS and IBC

The “intrinsic subtypes” of IBC (97) have been used to categorize DCIS, with an IHC approximation finding DCIS to be 49% Luminal A (ER+, Ki67 low), 8.7% Luminal B/HER2− (ER+, Ki67 high), 17% Luminal B/HER2+ (ER+, HER2+), 16% HER2 (ER−, HER2+), and 7% Triple Negative (ER−, PR−, HER2−) (98). These frequencies contrast with IBC where there is a higher proportion of triple negative (14–24%) and a lower proportion of HER2 (6–7%) (56, 99). Also in contrast to IBC, the subtypes may have limited prognostic value for DCIS, with one study showing only the triple-negative group having a worse long-term outcome (98), which was not statistically significant when adjusted for age, size, grade, and therapy. Another study found the Luminal A group to have a better survival in a multivariate analysis (100), but could not differentiate between the other groups. Interestingly, a study investigating different spatial areas of DCIS noted significant variability in subtyping with 35% showing more than one intrinsic subtype (30).

Several gene expression studies have been conducted for both pure and mixed DCIS. While the individual gene lists differ between studies, there are a number of common themes. First, both mRNA and microRNA profiling have found that the strongest expression differences are between normal epithelium and DCIS, rather than between DCIS and IBC (70, 101). Second, DCIS shows greater similarity to concurrent IBC than to other DCIS (102, 103), illustrating that inter-tumor heterogeneity is high and can mask more subtle changes. Expression profiles are strongly driven by intrinsic subtypes, and comparisons between not controlled DCIS and IBC are compromised when unmatched for tumor-intrinsic subtype (93). Nonetheless, studies of mixed and pure DCIS have found that differentially expressed genes between DCIS and invasive components commonly lie in pathways such as angiogenesis, cell–cell adhesion, epithelial-to-mesenchymal transition, and extracellular matrix (ECM) (70, 93, 102–104). However, differential expression of genes expressed highly in myoepithelial cells (e.g., SOX10) may merely indicate that the cells surrounding the epithelial DCIS tumor cells were included in the RNA extraction procedure (70, 104).

One study that had identified the instrinsic subtype as a major confounding factor in differential expression analysis performed subgroup tests and found that genes different between DCIS and IBC varied across subtypes (93). Luminal tumors were more likely to differentially express genes in adhesion and ECM pathways, HER2 tumors additionally had cell cycle pathways affected while basal-type tumors were more likely to have immune response genes affected.

Gene expression differences have also been observed between low- and high-grade DCIS, most commonly affecting cell growth and metabolism genes (105, 106). When these differentially expressed genes are applied to intermediate grade cases, many of these could be classified as either low or high grade (105), although Hannemann et al. identified a small group of intermediate grade DCIS that were not closely related to either high- or low-grade cases (106).

### Epigenetics of DCIS

Epigenetic analysis of DCIS has primarily been limited to single gene studies of promoter methylation, often with widely varying results depending on the method of detection and the analytical threshold to methylation positivity applied (107). Nonetheless, as with expression studies, some general points appear to be consistent. Increased levels of promoter methylation have been noted in the progression from normal epithelium to DCIS, but few studies show an increase in methylation in invasive progression and only for a subset of genes examined [e.g., *TWIST* 1 (108), *FOXC1* (109), *HOXA10* (110)]. A genome-wide methylation analysis could not discriminate in an unsupervised way between pure DCIS, mixed DCIS, and IDC (111). Methylation studies lag substantially behind other genome-wide approaches in terms of testing subgroups of DCIS, such as based on intrinsic subtyping, which could assist in teasing out subtle differences between DCIS and IDC.

Elevated levels of DNA methylation across multiple gene promoters have been associated with poor prognostic features such as high grade, HER2 positivity, and ER negativity (112), however, as yet only a single genome-wide study has undertaken an unbiased examination of the association of methylation with recurrence (113). This study identified significantly differentially methylated CpGs with enrichment for genes associated with homeobox regulation, limb morphogenesis, and polycomb target genes. Although the individual genes often differ, the methylation of homeobox genes is a recurrent feature of several methylation studies of DCIS, including three other genome-wide approaches (70, 114, 115).

## Progression of DCIS to Invasive Disease

There are a number of theoretical models for the development of DCIS and its progression to invasive disease, based on molecular profiling and animal studies (116). These models vary depending on the ER status of the tumors and also on the grade, whereby ER positive invasive carcinomas are thought to arise from ER positive precursors (such as ADH and DCIS), low-grade invasive cancers arise from low-grade DCIS and so on. The models may also be related to the putative cell of origin of each subtype, with different normal breast cells proposed to be the cells of origin for different invasive subtypes (117). The intermediate lesions may also be different, as may the length of time spent in each histological stage. For example, while basal-type invasive carcinomas are thought to arise from a luminal stem cell, they are not thought to progress *via* the hyperplasia–ADH–DCIS pathway, but to rapidly progress from an unknown but short-lived intermediate into high-grade DCIS and then quickly to invasive carcinoma. This model is supported by the relative underrepresentation of the basal/triple negative subtype in DCIS cohorts (73, 118). On the same basis, HER2 positive tumors are thought to remain for longer in a DCIS state before progressing. Basal and triple negative invasive tumors are also less likely to have a DCIS component, while HER2 positive invasive tumors have the most extensive associated DCIS (119). The biological mechanism for these differences is unclear, especially for the HER2 positive tumors, which are among the most aggressive of invasive subtypes.

The study of microinvasive DCIS may offer insights into the process and conditions under which invasion might occur. DCIS with microinvasion are more likely to be large, detected clinically rather than through mammographic screening and to show poor prognostic factors such as high grade, comedo necrosis and ER negativity, and have a worse outcome compared to DCIS without microinvasion (120, 121). At present, investigation of the molecular features of microinvasion has been limited to immunohistochemical analyses, in which it is clear that microinvasion is associated with alterations in the local microenvironment, both of the myoepithelial cell layer and the stromal cells (122, 123). However, it is not clear whether such changes are causative of, or reactive to, invasion. Future studies employing single-cell transcriptome or genome sequencing of the cells involved in a microinvasive event could enhance our understanding of the invasive process.

## The Relationship of Primary DCIS to Recurrences

Assessment of the clonal relationship between primary DCIS and later recurrent disease has been attempted in a number of different ways. Nuclear grade evaluation shows varying levels of concordance, with the same grade seen in 70–85% of recurrences when returning as DCIS and 49–53% when invasive (124, 125), although better concordance with invasive grade is seen when the nuclear pleomorphism component only is considered (76%). Immunophenotypic analyses of DCIS and recurrences have shown that recurrence as DCIS and invasive carcinoma both have high ER status concordance (85 and 84%, respectively, and 85–95% overall) as well as high HER2 concordance (88 and 91%, 88–89.5% overall) (124, 126). Immunostaining of p53 was also highly consistent [93% concordance (124)].

Using such immunohistochemical and pathological features, clonality could be estimated as anywhere between 50 and 95%. However, the levels of concordance observed by these parameters do not accurately represent the clonality rate as: (1) the assays can have poor reproducibility across time, with differing laboratory procedures and pathologist scoring leading to false non-clonality calls, (2) caveats to using grade include that intermediate grade has low inter-observer reproducibility and that overall invasive grade is measured differently to DCIS grade, and (3) most critically, measuring these common, low variability features is a blunt tool for positively assessing clonality: many tumors will share grade and ER status and be entirely independent tumors.

Genetic data have the potential to accurately determine clonality since recurrent tumors arising from remnants of the primary tumor will share key somatic driver events due to the shared ancestral origin. There is a surprising paucity of data on the genetic relationship of DCIS and their recurrences. Genome-wide copy number analysis showed that 6/8 recurrences within 5 years after treatment with WLE were clonally related to the primary tumor, and two cases had no copy number changes with shared breakpoints indicative of a clonal relationship (68). In an older, low-resolution copy number study 17/18 recurrences occurring within 10 years were clonally related to their primary tumor (127); however, no invasive recurrences were assessed and at least five of the cases had involved surgical margins and no RT, which increases the chance of clonal recurrence from residual disease. Consequently, the high level of concordance reported by this study may be an overestimate, and also not representative of invasive recurrences. A small microsatellite analysis of LOH found evidence for a clonal relationship in 3/3 DCIS recurring again as DCIS, even in one case after a 15-year interval (128). A second LOH analysis (129) evaluated a rare group of seven patients where DCIS was mis-diagnosed and left untreated, each later developing invasive carcinoma. Just three cases showed definite clonal relationship and one case was not clonally related, three cases were equivocal/uninformative.

The above genetic studies comprise a total of just 35 informative cases, mostly using low-resolution methodologies and with only a very few as recurrent invasive disease. The overall concordance rate is at best 31/35 (89%, binomial confidence interval 73–97%), but this could well be an underestimate, given the caveats described above.

## Biomarkers of Recurrence and Progression

Molecular biomarkers to predict recurrence after a DCIS diagnosis can be any of protein, RNA, or DNA molecules. To date, several studies have evaluated each in various DCIS cohorts; however, none are in clinical practice, mostly due to a combination of lack of validation in independent cohorts and/or low predictive value. The majority of studies performed are underpowered for accurate detection of predictive value (130, 131).

Protein biomarkers using IHC have been the most commonly assessed in DCIS, and as reviewed by Lari and Kuerer (131), many of the studies find associations with DCIS recurrence that are not validated by others (Table 2). However, some of the strongest candidates also supported by more recent studies include HER2, COX2, Ki67 (>10% positive cells), and p16.

**Table 2:** Summary of results of IHC studies of ductal carcinoma *in situ* (DCIS) recurrence reviewed in Lari and Kuerer (131).

Protein	No. studies significant	Total no. patients	Direction of association	Subsequent studies
COX2	4/4	629	Positive	Associated with metastases only (132)
Ki67	6/9	1,365	Positive	3 significant (25, 100, 133); 1 not significant (132)
HER2	4/14	2,365	Positive^a^	Significant for DCIS (25, 134)
ER	4/16	2,470	Negative	–
PR	2/13	2,051	Negative	–
p53	3/10	1,355	Positive^b^	2 not significant (132, 133)
p16	2/3	576	Positive	Significant (132)
Bcl-2	2/3	433	Negative	–
CD10	2/2	151	Contradictory	–
Cyclin D1	1/5	443	Negative	Not significant (133)
Cyclin A	1/2	110	Positive	–
p21	1/4	365	Positive	–
p27	0/2	237	ns	Not significant (135)
EGFR	0/2	288	ns	–
HER3	0/2	288	ns	–
HER4	1/2	288	Negative	–
VEGF	0/1	103	ns	–
MYC	0/1	159	ns	–
Survivin	1/1	161	Positive	Not significant (133)
AR	0/1	95	ns	–
SPARC	1/1	97	Positive^c^	–
Cyclin E	0/1	177	ns	–

*Ns, not significant*.

*^a^May only be associated with recurrence as DCIS*.

*^b^Scoring methodology for p53 highly variable*.

*^c^Stromal expression only*.

In one of the largest studies to date of multiple markers, Kerlikowske et al. (22), examining a 329 case cohort, identified different combinations of proteins to be predictive of recurrence as DCIS (ER-/HER2+/Ki67+ or COX2−/Ki67+/p16+) or IBC (COX2+/Ki67+/p16+). In combination with clinical factors (margin status for DCIS recurrence and method of detection for invasive recurrence), their stratification of the cohort into risk groupings identified low-risk groups (~4% chance of recurrence as each of DCIS and IBC within 8 years) and high-risk group (24 and 20% chance of recurrence as DCIS and IBC, respectively). These findings were partially validated by Rakovitch et al. (25), who also found HER+/Ki67+ (but not ER−) DCIS to be more likely to recur as DCIS, but were not predictive of IBC recurrence. However, they did not include COX2 or p16 in their panel of markers. A follow-up analysis by the Kerlikowske group with an additional 5 years of outcome data and 442 new cases added found that p16 positivity was associated with both local and regional/metastatic invasive recurrence (132). COX2 and Ki67 were not individually predictive, but COX2 positivity added value in prediction of metastatic recurrence. The highest risk group of regional/metastatic recurrence were p16+/COX2+/ER−/HER2+ (22.5% 10-year risk), but this comprised just 3% of the cohort.

Several independent studies have also found COX2 to be predictive of recurrence; however, most analyses did not differentiate between DCIS and IBC recurrence (136–138). One study only found COX2 positivity to be predictive of recurrence in combination with high Ki67 (139).

Two other studies have also found that tumors with p16+/Ki67+ have a higher risk of recurrence of either DCIS or IBC (139, 140). Interestingly, Witkiewicz et al. (*n* = 126, WLE only) also evaluated stromal p16 staining and found that high stromal expression was strongly correlated with disease recurrence. In particular, only a single case lacking stromal p16 expression had an invasive recurrence (140).

Thus far, only a single small study has evaluated copy number as a biomarker of recurrence, and found that DCIS that recurred were more likely to have increased levels of copy number change, with significant enrichment of gains of 20q and 17q, loss of chromosome 15 and allelic imbalance of chromosome 10 (68). However, these alterations have yet to be validated in an independent cohort. No studies have been performed to assess the association of mutations in DCIS with recurrence. One study found *GATA3* mutations to be present at a higher frequency in DCIS than invasive carcinoma and did not detect a difference in recurrence rates between mutated and wild-type tumors, but could not address invasive recurrences as none occurred in the evaluated cohort (69).

Gene expression by detection of mRNA is a popular approach in IBC, but to date only the OncotypeDX assay has been adapted for DCIS recurrence prediction. This 12-gene quantitative PCR-based assay (7 test and five control genes) has been tested in two cohorts (141, 142). Both found that the test had prognostic value in multivariate analyses (Table 3), and yet the low-risk group still had a 10-year chance of any recurrence of 10–13%. Neither study was able to demonstrate any difference in outcome between intermediate- and high-risk groups. There are limitations within the studies in that: only ~ 50% of patients in each cohort could be tested, which may bias the cohort; a result was not possible for ~15% of cases; the confidence intervals were very wide, approaching 40% (especially in intermediate and high-risk groups); and the follow-up was only ~10 years. Clinicopathological data were also incomplete on margin status and adjuvant treatment, both of which will influence outcome. In addition, the cases were drawn from a prolonged period (1994–2003) during which advances in surgical techniques have improved. In a subsequent study, Rakovitch et al. also evaluated the effect of RT on this predictive test (143). The low-risk group did not greatly benefit from the addition of RT, whereas the higher risk groups did benefit.

**Table 3:** Predictive value of OncotypeDX-ductal carcinoma *in situ* score.

	10-year risk any local recurrence		10-year risk local invasive recurrence	
Risk category	Wide-local excision (WLE) only	WLE + radiotherapy (RT)	WLE only	WLE + RT
Low	10–16%	9.4%	4–10%	6.8%
Intermediate	27–33%^a^	13.6%^a^	12–21%^a^	Not reported
High	26–33%	20%	16–19%	12%

*^a^Unadjusted*.

A small study comparing OncotypeDX with histopathological features suggested that a low score could be predicted by a combination of PR status, immune infiltrate, and mitotic count (144). Such a low-cost approach would be beneficial, especially given that economic modeling found no circumstances in which the OncotypeDX assay could be cost-effective in determining who should receive RT (145). However, incorporating existing risk parameters in concert with a molecular assay could improve the predictive benefit, and a small clinical utility assessment found that patient anxiety and decisional conflict were reduced after receiving assay results (146).

Interestingly, many of the risk factors associated with recurrence appear to be more strongly associated with recurrence as DCIS rather than invasive disease. For example, high grade was significantly associated with DCIS but not invasive recurrence in two recent biomarker studies (22, 25). HER2 positivity also is similarly more strongly related to DCIS recurrence. This feature was evaluated by Zhou et al. (147), who identified that DCIS recurring as IBC were more often ER positive, while DCIS recurrences were more often HER2 positive or EGFR positive. No difference was observed for Ki67, CK5/6, or PR. In multivariate analyses, symptomatic DCIS was more likely to recur as invasive disease than mammographically detected DCIS, but contrastingly, large tumor size was more often seen in tumors recurring as DCIS. Grade, margins, and treatment type were not different between invasive and non-invasive recurrences.

## DCIS Microenvironment and Relevance to Progression

The apparent molecular similarities between DCIS and invasive disease together with lack of detection of robust tumor-intrinsic biomarkers for invasive recurrence after DCIS (i.e., present in the tumor epithelial cells) suggests that the breast microenvironment could play a critical role in progression of DCIS to IBC. The microenvironment includes multiple cell types, including the myoepithelial cells that encircle the duct, the stromal fibroblasts, the vascular system, and the immune cells, as well as the duct/acini basement membrane. All components are likely to be important in restraining DCIS within the duct.

The myoepithelial cell layer is thought to provide both a physical barrier to expansion of the luminal epithelial cells into the stroma and also an active tumor suppressor role (148) through secretion of inhibitory molecules like protease inhibitors and ECM proteins (149). Gene expression profiling of myoepithlial cells obtained from DCIS samples showed extensive differences compared with normal myoepithelial cells, more so than when comparing the epithelial cells or fibroblasts (150), including chemokines CXCL12 and CXCL14. DCIS myoepithelial cells also show upregulation of some integrins leading to altered TGF-β signaling (151) and reduced ability to produce basement membrane proteins such as laminin and collagen IV (152, 153). Expression of αvβ6 integrin in the myoepithelium of DCIS was associated with recurrence, but this has yet to be validated. Some DCIS thus appear to have a more abnormal myoepithelial cell layer than others, but this has yet to be convincingly associated with invasive progression and biomarker utility.

The cross talk between the stroma and cancer cells appears to be an important feature of invasive progression, with invasive breast cancer-associated fibroblasts (CAFs) promoting progression of DCIS to invasive carcinoma in mouse models (148, 154). The mechanism of this tumor promotion is complex, involving metabolic support and pro-tumor inflammatory cytokine production (155). Stromal expression profiling has identified several differentially expressed genes between normal and mixed DCIS-associated stroma, including ECM genes and matrix metalloproteinases, but few differences between stroma found close to the DCIS component compared with stroma proximal to the IBC component (104, 156). In contrast, when stroma close to pure DCIS was compared with stroma from IBC cases, angiogenesis-related genes were more highly expressed in the IBC-related stroma (102). These contrasting data suggest that the stromal environment may be different for pure DCIS compared to mixed DCIS (157). The capacity of CAFs derived from pure DCIS cases to promote tumor growth does not appear to have been tested. To date, a myxoid stroma type (158), and stromal expression of p16 (140) and SPARC (159) have been associated with DCIS recurrence, but only in single studies.

The vascular microenvironment of DCIS appears to be altered compared to normal breast, and also different to IBC based on gene expression studies (102, 103). Within DCIS, two different vascular patterns have been observed: a “necklace” of vessels surrounding the DCIS-affected duct and a “diffuse stromal” pattern, where an increase in microvessels is seen in the surrounding stroma (160–162) with the necklace pattern associated with different levels of the angiogenic factor thymidine phosphorylase arising from the DCIS tumor cells (163). Neither pattern is commonly observed around normal ducts, and the stromal pattern is more frequently associated with negative prognostic features such as HER2 positivity, necrosis, higher grade or Ki67 staining and larger size. The incidence of each pattern varied widely depending on the study (22–80% for the necklace and 37–57% for stromal) likely reflecting both different methods and different proportions of tumor subtype in each cohort. The presence of the stromal pattern did not significantly predict recurrence, however, both cohorts evaluating this feature were small (135, 160).

The immune microenvironment is increasingly being explored in DCIS, with various studies enumerating tumor-infiltrating lymphocytes (TILs) or undertaking immunohistochemical analysis of different immune cell types. As in IBC, stromal TILs have been associated with poor prognostic features such as comedo necrosis, high grade, large size, and ER negativity (162, 164–168). Similar results have been observed with B-lymphocytes (CD19+, CD20+, or CD138+) (169). HER2 positivity (162, 165, 170) and *TP53* mutation (171) have also been associated with elevated TIL levels. A gene expression profiling approach to identify determinants of invasiveness independent of tumor subtype found that the most commonly differentially expressed pathway was the immune signature (93). Recently, a combined analysis of genetic events and TILs in DCIS found that DNA copy number aberration load was positively associated with TIL levels (171). This result was in striking contrast to IBC, where immune signatures by RNAseq are negatively correlated with aneuploidy (172). Thus, an altered interaction with the immune microenvironment, for example, through tumor immune-editing, may be critical in the evolution of invasive disease. Despite this possibility, association of immune cells with recurrence has proven mixed. The largest analysis to date did not find any significant association of TILs with DCIS recurrence, although there were hints that different subtypes could have a different interaction between immune cell presence and recurrence (165). A more complex, but smaller, IHC study of immune cells determined that the type of immune cells present was critical to predicting recurrence, with CD8, HLADR, and CD115 being predictors (164). Thus, using the immune microenvironment as a predictive biomarker may be complex, requiring detection of specific immune cells in specific DCIS subtypes, with different biomarkers for IBC and DCIS recurrence, and needing to incorporate other features, possibly including genetic events.

## Conclusion

The biology of DCIS is still not well understood, and previous attempts have been compromised by underestimating the complexity and heterogeneity of the disease. As in IBC, DCIS is not a single disease, but varies based on hormonal status, growth factor receptor status, proliferation rate, and genetic features. In particular, the interaction of all these factors with the microenvironment in the initiation of neoplasia and in progression to invasive disease needs to be better elucidated. Biomarker studies will require integration of tumor-intrinsic factors (genetic events, intrinsic subtypes, proliferation rate, grade), tumor-extrinsic tissue factors (the immune response, stromal complexity, the relationship with the myoepithelium) and clinical factors (margins, tumor size, detection modality, patient age, treatment type, etc.), to be truly effective at predicting patient outcome and optimizing treatment. Advances in technologies enabling single-cell analyses will assist in developing our understanding of DCIS clonal heterogeneity and progression, while novel high-throughput proteomic approaches and multiplex spectral imaging assays will facilitate integrated analysis of multiple cellular phenotypes to be interrogated, maximizing the information that can be obtained from limited material. In addition, the proportion of DCIS with non-clonal recurrence needs to be assessed. Such complexity demands a collaborative, multicenter approach to have sufficient statistical power for biomarker validation and implementation. Recently, the Cancer Research UK Grand Challenge and the Dutch Cancer Society funded a GBP15 million study to analyze DCIS for biomarkers, and the Australian-led PRECISION study has similar goals, indicating that funders are now recognizing the need to invest in large-scale projects to tackle the issue.

## Author Contributions

KG and SF conceived of and drafted the manuscript and figures and gave final approval of the version to be published.

## Conflict of Interest Statement

The authors declare that the research was conducted in the absence of any commercial or financial relationships that could be construed as a potential conflict of interest.
